# A New Surface Plasmon Resonance-Based Immunoassay for Rapid, Reproducible and Sensitive Quantification of Pentraxin-3 in Human Plasma

**DOI:** 10.3390/s140610864

**Published:** 2014-06-19

**Authors:** Mara Canovi, Jacopo Lucchetti, Matteo Stravalaci, Sonia Valentino, Barbara Bottazzi, Mario Salmona, Antonio Bastone, Marco Gobbi

**Affiliations:** 1 Department of Biochemistry and Molecular Pharmacology, IRCCS-Istituto di Ricerche Farmacologiche “Mario Negri”, Milan 20156, Italy; E-Mails: mara.canovi@gmail.com (M.C.); jacopo.lucchetti@marionegri.it (J.L.); matteo.stravalaci@marionegri.it (M.S.); mario.salmona@marionegri.it (M.S.); antonio.bastone@marionegri.it (A.B.); 2 Humanitas Clinical and Research Hospital, Rozzano, Milan 20089, Italy; E-Mails: Sonia.Valentino@humanitasresearch.it (S.V.); Barbara.Bottazzi@humanitasresearch.it (B.B.)

**Keywords:** surface plasmon resonance, SPR-based immunoassay, pentraxin-3, plasma, sepsis

## Abstract

A new immunoassay based on surface plasmon resonance (SPR) for the rapid, reproducible and sensitive determination of pentraxin-3 (PTX3) levels in human plasma has been developed and characterized. The method involves a 3-min flow of plasma over a sensor chip pre-coated with a monoclonal anti-PTX3 antibody (MNB4), followed by a 3-min flow of a polyclonal anti-PTX3 antibody (pAb), required for specific recognition of captured PTX3. The SPR signal generated with this secondary antibody linearly correlates with the plasma PTX3 concentration, in the range of 5–1500 ng/mL, with a lowest limit of detection of 5 ng/mL. The PTX3 concentrations determined with the SPR-based immunoassay in the plasma of 21 patients with sepsis, ranging 15–1600 ng/mL, were superimposable to those found in a classic ELISA immunoassay. Since the PTX3 concentration in the plasma of healthy subjects is <2 ng/mL, but markedly rises in certain medical conditions, the method is useful to quantify pathological levels of this important biomarker, with important diagnostic applications. In comparison with the classic ELISA, the SPR-based approach is much faster (30 min *versus* 4–5 h) and could be exploited for the development of new cost-effective SPR devices for point-of-care diagnosis.

## 1. Introduction

The long pentraxin-3 (PTX3) is a member of the pentraxin family originally identified as an early induced gene in endothelial cells and macrophages [1]. The prototype of the pentraxin family is C-reactive protein (CRP), a pentameric molecule produced systemically by the liver in response to IL-6 and widely used as a biomarker for human pathologies involving acute immunological responses [2]. PTX3 is an octameric protein with a CRP-like pentraxin domain and a long, unrelated N-terminal domain. Unlike CRP, PTX3 is produced by various cells in peripheral tissues, e.g., macrophages, dendritic cells, endothelial cells, smooth muscle cells and fibroblasts, in response to primary proinflammatory cytokines (IL-1 and TNFα) and microbial recognition [3].

PTX3 blood levels are ≤2 ng/mL in healthy subjects [4–6] but rise rapidly (peaks at 6–8 h) in acute myocardial infarction (AMI) to 3–5 times the normal range [5,7]. Even more dramatic increases in plasma PTX3 (up to 1500 ng/mL) are observed during endotoxic shock, sepsis, and other inflammatory and infectious conditions, including *A. fumigatus* infection, meningococcal diseases, dengue, tuberculosis, and leptospirosis [6,8–11]. In a small group of critically ill patients with systemic respiratory distress syndrome, sepsis or septic shock, PTX3 levels correlated with the severity of the disease and infection [12–14], suggesting that PTX3 could be a candidate prognostic marker and might be associated with the risk of mortality in severe sepsis and septic shock [13,15,16].

Plasma PTX3 is currently conveniently detected by sandwich ELISA [5] which, however, requires several different steps and takes a relatively long time to give results. In this study, we aimed to develop and characterize a surface plasmon resonance (SPR)-based immunoassay to quantify plasmatic PTX3 in a faster and simpler way than classic ELISA. SPR is an optical phenomenon which occurs when polarized light, under conditions of total internal reflection, excites a surface plasmon wave at the interface between a highly conductive metal and the thin dielectric film forming on the metal surface. It is widely used to characterize the interaction between two unlabeled molecules, one immobilized on a sensor chip, and the other flowed through a microfluidic system over the chip surface. Binding is measured in real time as a change in the effective refractive index of the surface-bound biofilm, which affects the coupling conditions of incident light with surface plasmon.

The most common application of SPR instruments is to determine affinity parameters for biomolecular interactions [17], but its versatility allows many other uses [18–22]. These include label-free immunoassays [23] using antibodies immobilized on the sensor chip as sensing elements for detection of the corresponding antigen, in real time and with high sensitivity and selectivity. Thus, in the present study we immobilized an anti-PTX3 antibody on the sensor chip and assessed its ability to recognize flowing PTX3, in either buffer or plasma, to quantify plasma PTX3. We also used a more specific sandwich approach, possible with SPR protocols, in which the PTX3 captured by the immobilized antibody is directly recognized and quantified by flowing a secondary anti-PTX3 antibody.

## 2. Experimental Section

Recombinant human PTX3 (rhPTX3) and the antibodies against human PTX3 (MNB4 and pAb) have been described elsewhere [5,24]. Briefly MNB4 is a rat monoclonal antibody generated immunizing rat with human recombinant PTX3 while pAb is rabbit polyclonal antiserum raised against human PTX3 and purified by immunoaffinity. We used plasma from healthy subjects and patients diagnosed with sepsis. The study was approved by the ethics committee of the Istituto Clinico Humanitas, Rozzano, Italy. Blood samples were collected into ethylenediaminetetraacetic acid (EDTA). Tubes were centrifuged and plasma stored at −70 °C. Samples were thawed and divided into aliquots for analysis.

### 2.1. SPR-Based Immunoassay for PTX3

For SPR interaction studies, we used a ProteOn XPR36 (BioRad, Hercules, CA, USA) apparatus, which has six parallel flow channels that can immobilize up to six ligands on the sensor chip surface. After ligand immobilization, the ProteOn XPR36 fluidic system can rotate 90° [25], so that up to six different analytes can be injected simultaneously over the ligands.

**Figure 1.**
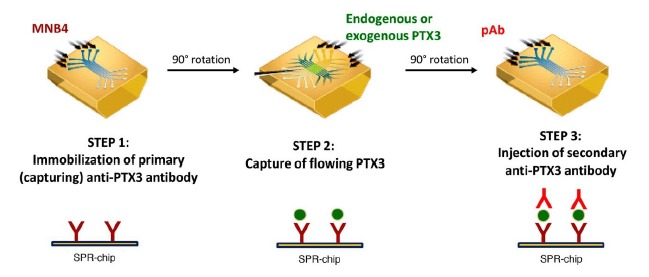
Schematic representation of SPR-based immunoassay for PTX3 quantification. The immunoassay is based on three consecutive steps: (1) immobilization of the primary anti-PTX3 antibody (MNB4), (2) capture of PTX3, (3) specific recognition of the captured PTX3 by the secondary anti-PTX3 antibody (pAb).

The final procedure comprised in three consecutive steps (Figure 1): (i) immobilization of the primary monoclonal anti-hPTX3 antibody (MNB4), usually in a single flow channel of the sensor chip; (ii) 90° rotation and injection of up to six PTX3-containing samples over the immobilized MNB4; and (iii) 90° rotation and injection of a secondary polyclonal anti-hPTX3 antibody (pAb) in the flow channel initially coated with MNB4.

MNB4 was immobilized using amine-coupling chemistry on the surface of a GLC sensor chip (BioRad), with a modified alginate polymer bound to a gold surface. Briefly, surface was activated with sulfo-N-hydroxysuccinimide/1-ethyl-3-(3-dimethyilaminopropyl)-carbodiimide (sulfoNHS/EDC) according to manufacturer's recommendation. MNB4 (30 μg/mL in acetate buffer, pH 5.0) was then flowed for 5 min at a rate of 30 μL/min, and the remaining activated groups were blocked with ethanolamine, pH 8.0. The final immobilization level was about 4000 resonance units (RU, where 1 RU = 1 pg·protein/mm^2^). In preliminary studies, the same procedure was used to immobilize the same amount of an antibody against C-Reactive Protein (CRP) in a parallel channel, as reference. After rotation of the fluidic system, samples (buffer spiked with rhPTX3 or human plasma samples containing added rhPTX3 or endogenous PTX3) were flowed for 5 min at a rate of 30 μL/min. 10 mM phosphate buffer containing 150 mM NaCl and 0.005% Tween 20 (PBST, pH 7.4) was used. Before injection, plasma samples were incubated for 10 min with 0.05% polybrene to reduce the interaction of PTX3 with plasma protein, and diluted five-fold in PBST. This dilution gave the best compromise between the need to reduce the plasma-dependent bulk of the SPR signal and the need to maintain sufficient sensitivity of the assay. The fluidic system was then restored to the initial orientation (Figure 1) and after 20-min washing the pAb antibody was injected at the concentration of 15 μg/mL, for 3 min at a flow rate of 30 μL/min.

After each three-step session, the sensor surface was regenerated by short pulses of 10 mM glycine-HCl buffer (pH 2.5) (18 s at a flow rate of 100 μL/min), to allow us to repeat analysis on the same chip. In our experience, we could regenerate twice.

The sensorgrams (time course of the SPR signal in RU) were normalized to a baseline value of 0. Parallel injections of vehicle alone allowed correction for binding-independent responses (*i.e.*, drift effects).

### 2.2. Enzyme-Linked Immunosorbant Assay (ELISA)

A sandwich ELISA assay was set up to measure PTX3 levels in human plasma samples. Briefly, 96-well ELISA plates (Nunc MaxiSorp, Thermo Fischer Scientific, Roskilde, Denmark) were coated with 100 ng of rat monoclonal antibody MNB4 in 100 μL of coating buffer (15 mM carbonate buffer pH 9.6) and incubated overnight at 4 °C. After each step plates were washed three times with washing buffer (PBS containing 1.17 mM CaCl_2_, 1.05 mM MgCl2 and 0.05% Tween 20, pH 7.00). To block non-specific binding sites, 300 μL of 5% dry milk in washing buffer were added and incubated for 2 h at room temperature. Fifty μL of either purified human recombinant PTX3 standards (75 pg/mL to 2.4 ng/mL, diluted in RPMI 1640 containing 2% bovine serum albumin), or plasma were then added in triplicate to each well and incubated for 2 h at 37 °C. After washing, 100 μL of biotin-conjugated pAb (5 ng/well) diluted in washing buffer were added. The plates were incubated for 1 h at 37 °C, washed extensively and incubated with 100 μL of Streptavidin-horseradish peroxidase (Biospa, Milan, Italy) diluted 1:4000. After 1 h incubation at room temperature, the plates were washed extensively before the addition of 100 μL of tetramethylbenzidine substrate (Thermo Fischer Scientific, Rockford, IL, USA). Reaction was blocked by addition of 50 μL of 2N sulphuric acid and absorbance was measured at 450 nm with an automatic ELISA reader. No cross reaction of MNB4 and pAb with human CRP and serum amyloid P component protein has been observed. Mean PTX3 content was calculated converting Abs450 values to protein concentration by means of the standard curve with recombinant purified human PTX3. Detection limit of our assay is 100 pg/mL and the interassay variability ranges from 8% to 10% [5].

## 3. Results

### 3.1. rhPTX3 Binding to Immobilized MNB4 in Buffer

**Figure 2.**
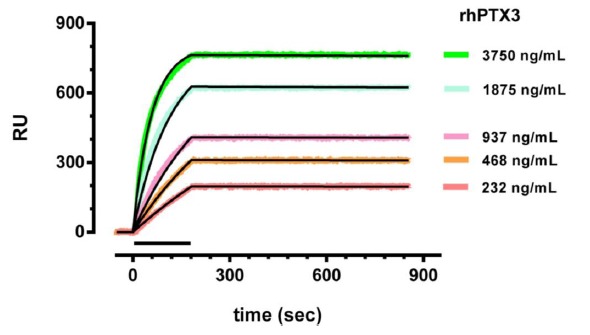
Binding of rhPTX3 to MNB4, in buffer. Different concentrations of rhPTX3 were injected for 3 min (bar), at a flow rate of 30 μL/min, over MNB4 immobilized on the sensor chip surface. The running buffer (also used to dilute rhPTX3) was 10 mM phosphate buffer containing 150 mM NaCl, added with 0.005% Tween 20 (PBST, pH 7.4). Sensorgrams were obtained after subtraction of the nonspecific response measured in parallel in surface immobilizing anti-CRP antibody (used as reference) and after correction for the signal when vehicle was injected alone. The curves were analyzed by the Langmuir equation, modelling a simple bimolecular interaction, and the fittings are shown in black. Data are expressed as resonance units (RU) against time.

Initial experiments were carried out in buffer (PBST) to verify the high-affinity binding between rhPTX3 and MNB4 covalently immobilized on the sensor chip. We injected five different concentrations of rhPTX3 (234–3750 ng/mL, corresponding to 0.625–10 nM octameric protein), or vehicle alone, simultaneously in six parallel lanes (“one-shot kinetics” approach [25]). In parallel, the same concentration of rhPTX3 also flowed onto a reference surface immobilizing an antibody against CRP. There was a concentration-dependent binding of rhPTX3 to MNB4 (Figure 2), and no binding to the anti-CRP antibody (data not shown). The fitting of the normalized sensorgrams (Figure 2), using the simplest 1:1 interaction model (Langmuir equation, ProteOn Analysis software, Bio-Rad Laboratories), confirmed very high-affinity binding, with an estimated KD of 4.3 ± 0.7 pM (calculated on octameric protein) and very slow dissociation rate constants (8.1 × 10^−6^ s^−1^). The lowest detectable rhPTX3 concentration was 15 ng/mL.

### 3.2. rhPTX3 Binding to Immobilized MNB4 in Plasma

**Figure 3.**
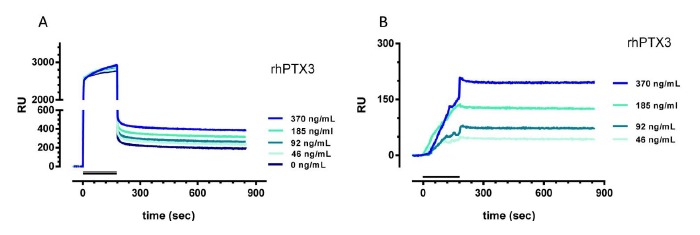
Binding of rhPTX3 to MNB4, in plasma. (**A**) Plasma of healthy subjects was spiked with different concentrations of rhPTX3 (0–370 ng/mL), diluted five-fold in PBST, and injected for 3 min (bar) at a flow rate of 30 μL/min, over the immobilized MNB4. (**B**) Normalized sensorgrams after subtraction of the signal with unspiked plasma. The running buffer was PBST, pH 7.4. Data are expressed as resonance units (RU) against time.

Plasma samples from healthy subjects, containing negligible levels of PTX3 (<2 ng/mL), were spiked with known amounts of rhPTX3, to obtain different concentrations (46–370 ng/mL are shown in Figure 3A), diluted five times with PBST and then flowed over the surface immobilizing MNB4. The unspiked diluted plasma gave a very high SPR signal, most of it (about 2500 RU) with fast association and fast dissociation (Figure 3A) hence very likely due to non-specific adsorption of plasmatic proteins on the sensor surface (plasma-dependent bulk signal). Some signal, about 200 RU, however had slower dissociation and was still present after 10 min of washing, indicating a stronger interaction with the coated surface. This interaction was non-specific because very similar bulks were found on CRP-coated and empty surfaces (data not shown). The presence of rhPTX3, however, caused a concentration-dependent increase of the SPR signal, which could be seen better after subtraction of the signal with unspiked plasma (Figure 3B). The normalized sensorgrams indicated that the rhPTX3-dependent binding to MNB4 was quantitatively similar to that in buffer (compare with Figure 2) and confirmed its very slow dissociation rate. The lowest detectable rhPTX3 concentration was about 20 ng/mL.

### 3.3. SPR-Based Immunoassay for PTX3 in Plasma

**Figure 4.**
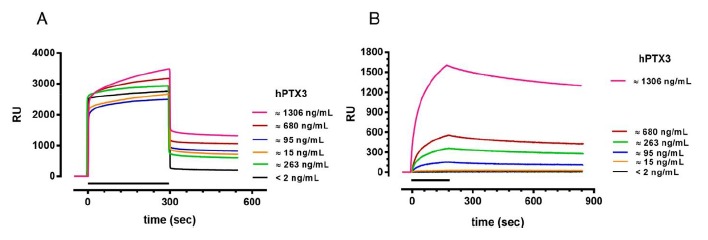
SPR-based immunoassay for PTX3 detection in human plasma. (**A**) Sensorgrams obtained injecting plasma samples, diluted five-fold with PBST, from subjects with different levels of endogenous PTX3 (determined by a classical ELISA) on the MNB4 immobilized on the sensor chip. Samples were injected for 5 min (bar) at a flow rate of 30 μL/min. (**B**) Sensorgrams obtained injecting a secondary anti-PTX3 antibody over the surfaces previously injected with plasma samples to highlight the PTX3-dependent binding signal. Samples were injected for 3 min (bar) at a flow rate of 30 μL/min. The running buffer was PBST, pH 7.4. Data are expressed as resonance units (RU) against time.

Although the SPR protocol described above detects rhPTX3 spiked in plasma (Figure 3), it did not prove suitable to quantify endogenous PTX3 present in patients' plasma (Figure 4A). When we injected diluted plasma samples from patients with different PTX3 levels and one patient with undetectable PTX3 (as determined by a classical ELISA assay) we found no correlation between the SPR signal and the PTX3 concentration. This is probably because the PTX3-independent SPR signal, highlighted with plasma samples without PTX3, masks the PTX3-dependent signal. The PTX3-independent SPR signal differed widely among subjects (see the samples with very low PTX3 levels, <2 and 15 ng/mL, Figure 4A) and as it is not known it cannot be subtracted to estimate the PTX3-dependent signal, as shown in Figure 3. Moreover, we did not succeed in obtaining reliable PTX3-dependent responses by subtracting the PTX3-independent signal obtained in parallel “reference surfaces”, e.g., activated/deactivated surface without any ligand or immobilizing different antibodies (data not shown). The reason is that the PTX3-independent binding signal occurring in these “reference surfaces” is different from the PTX3-independent binding signal occurring on the surface coated with MNB4, and thus it is not suitable for an effective subtraction.

These difficulties prompted us to add a further step after the injection of plasma samples, with the injection of a secondary anti-PTX3 antibody (pAb), to specifically recognize the PTX3 captured by MNB4. This protocol gave SPR signals which correlate with the endogenous levels of PTX3 (Figure 4B); no SPR signal was observed for samples from healthy subjects (PTX3 < 2 ng/mL, black sensorgram in Figure 4B) indicating the specificity of the signal generated with the pAb.

**Figure 5.**
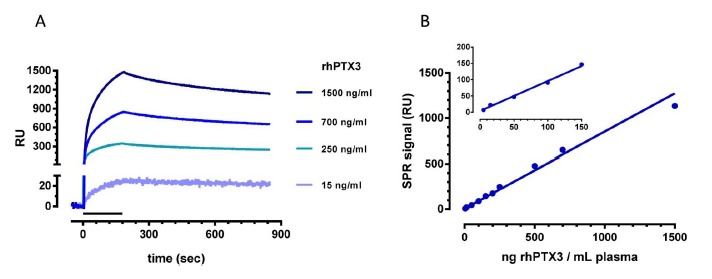
Linear correlation between the concentration of rhPTX3 and the SPR signal. Plasma of healthy subjects was spiked with different concentrations of rhPTX3 (5–1500 ng/mL), diluted five-fold in PBST, and injected for 5 min over the MNB4 immobilized antibody. Then, the pAbwas flowed for 3 min (bar) at a flow rate of 30 μL/min, over captured rhPTX3 and representative sensorgrams are shown in Panel **A**. Binding responses were subtracted of the signals detected on an empty surface, correcting for binding-independent responses such as bulk effects due to buffer exchanges or drift. (Panel **B**) Calibration curve obtained plotting plasma rhPTX3 concentrations against the SPR signal at the end of the 10-min dissociation phase. The inset highlights the SPR signal obtained at the lower rhPTX3 concentrations.

To establish the limit of detection and the range of linearity of this SPR-based sandwich protocol better, we injected plasma samples from healthy subjects, spiked with known amounts of rhPTX3 protein, from 5 to 1500 ng/mL. Figure 5A shows the sensorgrams obtained when flowing the pAb. Figure 5B shows the linear correlation between the concentration of rhPTX3 and the SPR signal measured at the end of the 10-min dissociation phase (Pearson r = 0.9916, *p* < 0.0001). The lowest detectable rhPTX3 concentration was 5 ng/mL plasma, resulting in a signal of about 7 RU (*i.e.*, three times the noise SPR signal). The data in Figure 5 were obtained in different experimental sessions, indicating the good reproducibility of the method. The inter-assay coefficient of variation was 11% with accuracy (relative error) 5%–19%.

Finally, we applied the SPR-based immunoassay to analyze 21 plasma samples from septic patients whose PTX3 levels, previously determined by a classic ELISA assay, ranged widely, from 15 to 1600 ng/mL. These plasma samples were diluted five-fold and injected over immobilized MNB4. Each injection session always included 4–5 patients' plasma samples and 1–2 “standards” (*i.e.*, plasma samples from healthy subjects spiked with a known amount of rhPTX3), for internal quality control. Soon after, we injected pAb and the corresponding SPR signal was used to quantify PTX3 concentrations, by interpolation from the calibration curve (Figure 5B).

**Figure 6.**
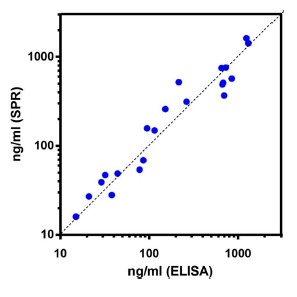
Correlation between plasma PTX3 levels quantified with the new SPR-based immunoassay and the traditional ELISA assay. Each point represents a single patient.

The correlation was highly significant between the plasma PTX3 levels determined with the new SPR-based approach and those determined with the classic ELISA immunoassay (linear correlation between log-transformed concentrations, Pearson r = 0.974, *p* < 0.0001) (Figure 6). The slope of the linear regression was 1.01 ± 0.05, indicating that the two methods gave superimposable results. The deviations from the identity line are probably due to experimental error in either the SPR-based immunoassay or the ELISA immunoassay, or both.

## 4. Discussion

We have developed and characterized a new SPR-based immunoassay for the determination of PTX3 levels in human plasma. The method is based on the capture of plasma PTX3 by an anti-hPTX3 antibody immobilized on the sensor chip surface (monoclonal MNB4 in our protocol), and the consequent recognition of captured PTX3 by a secondary polyclonal anti-hPTX3 antibody (pAb). The SPR signal generated when flowing the pAb linearly correlated with the concentration of plasma PTX3, in the range of 5–1500 ng/mL. Five ng/mL was the lowest limit of detection observed with the described experimental conditions, although it might be anticipated that longer injections of plasma samples would decrease this limit. The concentrations of PTX3 determined with the SPR-based immunoassay in the plasma of 21 septic patients were superimposable on those given by a classic ELISA. PTX3 levels in healthy subjects is <2 ng/mL [4–6], thus below the limit of detection of the SPR assay, but rises steeply in different pathological conditions to levels readily detected by the SPR immunoassay (see Introduction), which can therefore distinguish between healthy and diseased patients.

The new assay is essentially based on the same approach as the classic ELISA, with a capturing primary antibody immobilized on a solid phase and a secondary antibody to specifically recognize the antigen of interest. In theory, an SPR assay might not require the secondary antibody, since it may directly recognize the antigen bound to the immobilized antibody (hence the antigen concentration) as a change of mass at the sensor surface. This would substantially cut the experimental time but unfortunately it did not work for plasma PTX3. Despite the high affinity between rhPTX3 and the immobilized MNB4 (in the picomolar range, as assessed in buffer), we observed a significant plasma-dependent but PTX3-independent SPR signal which could not be quantified in “real” samples, thus preventing determination of the PTX3-dependent signal, and, consequently, the plasma PTX3 levels. This is a fundamental point which always needs to be considered, sometimes overlooked by proponents of new analytical assays/sensors who limit their studies to “proof-of-principle” data obtained by measuring the signal due to the exogenous analyte spiked in control plasma. We successfully circumvented the limitation by flowing, soon after plasma injection, the secondary anti-hPTX3 antibody (pAb) which allowed specific recognition of the captured PTX3. However, even with this step the SPR immunoassay took significantly less time than the classic ELISA. The latter needs longer incubation times with plasma and the secondary antibody (hours instead of minutes), additional step(s) for detection of the secondary antibody (e.g., streptavidin-HRP and substrate), as well as washings between the different steps. Thus, the whole procedure to detect and quantify pathological PTX3 levels (without considering the time needed to coat the solid phase with the primary antibody) takes less than 30 min with the new SPR immunoassay and 4–5 h with ELISA.

Although ELISA assays are more sensitive and cheaper at the moment, cost-effective SPR-based devices, employing pre-coated disposable chips and suitable for point-of-care diagnosis, may well be developed in the near future. The prototype of a new stand-alone, portable SPR-based imaging system has recently been described with proof-of-concept for detection of PTX3 [26]. Our data, with the characterization of the SPR protocols allowing sensitive and reproducible detection of pathological PTX3 levels in human plasma, offers the basis for further improvement of these devices, with very important diagnostic applications.

## 5. Conclusions

We have characterized a new SPR-based immunoassay for the rapid, reproducible and sensitive determination of PTX3 levels in human plasma, suitable to quantify pathological levels of this important biomarker, with important diagnostic applications. In comparison with the classic ELISA, the SPR-based approach is much faster and could be exploited for the development of new cost-effective SPR devices for point-of-care diagnosis.
